# The difficult conversation: a qualitative evaluation of the ‘Eat Well Move More’ family weight management service

**DOI:** 10.1186/s13104-018-3428-0

**Published:** 2018-05-21

**Authors:** Rebecca E. Johnson, Oyinlola Oyebode, Sadie Walker, Elizabeth Knowles, Wendy Robertson

**Affiliations:** 1 Collaboration for Leadership in Applied Health Research and Care West MidlandsCollaboration for Leadership in Applied Health Research and Care West Midlands, Division of Health Sciences, Warwick Medical School, University of Warwick, Coventry, CV4 7AL England, UK; 2 Public Health and Commissioning, Solihull Metropolitan Borough Council, Council House, Manor Square, Solihull, B91 3QB England, UK

**Keywords:** Knowledge exchange, Public health practice, Health communication, Qualitative, Child obesity

## Abstract

**Objective:**

The Eat Well Move More (EWMM) family and child weight management service is a 12-week intervention integrating healthy eating and physical activity education and activities for families and children aged 4–16. EWMM service providers identified low uptake 12 months prior to the evaluation. The aims of this study were to describe referral practices and pathways into the service to identify potential reasons for low referral and uptake rates.

**Results:**

We conducted interviews and focus groups with general practitioners (GPs) (n = 4), school nurses, and nursing assistants (n = 12). Data were analysed using thematic analysis. School nurses highlighted three main barriers to making a referral: parent engagement, child autonomy, and concerns over the National Child Measurement Programme letter. GPs highlighted that addressing obesity among children is a ‘difficult conversation’ with several complex issues related to and sustaining that difficulty. In conclusion, referral into weight management services in the community may persistently lag if a larger and more complex tangle of barriers lie at the point of school nurse and GP decision-making. The national prevalence of, and factors associated with this hesitation to discuss weight management issues with parents and children remains largely unknown.

## Introduction

Childhood obesity has short and long-term consequences for physical and mental health [1, 2]. It is recognised by the World Health Organisation as one of the most serious public health challenges of the 21st century [3]. Despite more than a decade of policy attention, a high prevalence of childhood obesity persists in the UK [4]. For those children who are overweight, behavioural lifestyle interventions can result in clinically meaningful reductions in overweight in children and adolescents, compared to standard care or self-help [5–7].

In England, the National Institute for Health and Care Excellence (NICE) recommends that tailored clinical interventions should be considered for children with a body mass index (BMI) at or above the 91st centile [5–8]. Public Health teams, situated within local government, typically commission these services. Over 300 of these services are likely to be running in England [9]. NICE guidance does not specify who should identify and refer eligible children into provided services and these practices may vary.

Many eligible children do not benefit from existing services. This is partly due to attrition, where a child enters but does not complete a programme, reported to be between 27 and 90% [10]. However, many eligible children may not be referred to appropriate services, or may be referred but never initiate treatment. While there is a growing literature on attrition, research into barriers to referral to, and initiation of, childhood obesity treatment remains scant [11].

This qualitative study explores the challenge surrounding low referral and uptake rates into a community child weight management programme despite comparatively high retention, completion and service satisfaction of participants. The study objectives were to (a) describe current referral practices and pathways into the programme, (b) identify potential reasons for low uptake, and (c) make recommendations to improve service referral.

## Main text

### Methods

#### Intervention

The intervention “Eat Well Move More” (EWMM) combines healthy eating and cooking education with physical activity sessions. Three service offers exist: a school programme (4–16 years), community programme (7–11 years) and one-to-one sessions (12–16 years). The intervention was developed in Solihull, England using Public Health Outcome Framework guidance, and principles of behaviour change [12, 13]. Children may be referred to EWMM by general practitioners (GPs), school nurses, family support workers, paediatricians, or self-referral via the National Childhood Measurement Programme (NCMP). NCMP measures height and weight of school children in England. A letter is sent to families indicating the child’s weight status based on BMI [14]. Information on EWMM and healthy lifestyles is provided with the letter, and parents can self-refer into EWMM on this basis. EWMM allows rolling admissions, so families do not have to wait to join. It is free for referred children.

#### Data collection

Qualitative, semi-structured interviews and focus groups (FGs) were conducted with GPs and school nurses from November 2015 to March 2016, by two female researchers with previous interview experience and no previous relationship with participants (RJ/WR). Topic guides included questions about referral into EWMM and invited responses on any other aspect of EWMM. Interviews and FGs were audio recorded, and were an average length of 10 min per interview and 30 min per FG.

Purposive sampling was used to request participation. All GP practices within Solihull (N = 36) were contacted first by telephone, second by email if listed and third by fax. Targeted calls to practices where GPs were known to refer into EWMM were completed as a second wave of recruitment. Personalised emails from a Public Health Consultant were completed as a third wave of recruitment. Face-to-face or telephone interviews were offered to GPs to suit their schedules.

School nurses were recruited via email and telephone with all local area school nurse leads (N = 2); a request was sent to attend school nurse monthly meetings. FGs were conducted at school nurses’ monthly meetings to maximise the range of views collected. Nurses attended from schools in deprived and affluent areas of the community to reflect socio-economic disparity in the prevalence of child obesity [15, 16].

#### Data analysis

Data were transcribed verbatim and anonymised. Each dataset was analysed using a thematic analysis approach [17]. RJ coded all data initially and these codes were cross-checked and discussed with WR to ensure fit. Data were organised in NVivo [18]. Data were analysed deductively. Interviews and FG transcripts underwent initial and then axial coding. Categories were identified and themes emerged through an iterative process of refining and expanding emerging concepts and issues related to the research questions.

### Results

Interviews and FGs completed or attempted are described in Table 1.

**Table 1:** Participant groups and numbers of evaluation participants

Evaluation Group	Number of participants (observed)	Number of participants attempted	Method of collection	Duration average (min)
General Practitioners	N = 4 4 GPs from 4 different general practices	36 general practices 90 attempts to contact (Lack of time given as main reason for non-participation)	Telephone interview (4)	10
School Nurses and Nursing Assistants	N = 12	N = 12	Focus group (2)	30

Two FGs were conducted with School Nurses and Nursing assistants and four GPs completed interviews. Findings are detailed below.

#### School nurses’ views

Most school nurses described an awareness of EWMM, yet only three had made referrals into EWMM. Barriers to pupils being successfully referred to the EWMM programme emerged from school nurses’ experiences which are reflected in three themes (1) parent engagement; (2) children’s autonomy; (3) NCMP letter (Box 1).

#### Parent engagement

School nurses discussed how parents acted as barriers and facilitators to EWMM referrals. Nurses described scenarios where children sought out the school nurse to address their weight, ending with parent contact for permission to refer the child into EWMM. The referral would not then be made because the parent declined the referral. No further action would be taken on behalf of the child. Direct quotes reflect some of the issues Nurses expressed relating to this scenario (Box 1).

##### Box 1: School nurses’ views and experiences

**Table:** 

Topic	Quotation
Parent Engagement with School Nurses Parents as a barrier to referral	*“But I’ve said to parents, just from asking for consent, to just speak to Eat Well Move More and see what they’re about and just have a bit more information about it. But they don’t want it, it’s just you get completely shut down”* *“[School Nurse relaying conversation with parent] “I think we’ll sort it out on our own because he’s doing a bit more exercise, he’s cutting down on the crisps and stuff”…So that’s it, it stops”* *“But you’re failing that child in a way aren’t you. And the referral to Eat Well is stopped because there’s no parental consent, so you can’t… we see them in drop ins and things and healthy plate and healthy eating and give them the leaflets, but…”*
Challenge of engaging parents and children as a unit	*“SN2: I think the issue with that is though, it’s no good just having the children, you do need parents on board as well, and then they’re going through the same process really”* *“SN1: Well I think the children probably would have more impact on the parents than we would”*
Parents as facilitators	*“But again it’s hard because that child is not… or young person is not in control of the cooking at home. And I’m guessing that along with overweight children you’ve probably got overweight parents, so it’s not just about impacting that one child it’s about trying to get the whole family on board”*
Child autonomy	*“But I think they come very positive and want some help, but I think it’s then their ability to take that forward, which I suppose is about the adolescence and, you know, whether their brain works really about they want to do it but they can’t”* *“Yeah, they’re thinking about body shape and how they feel about themselves”* *“meals at home aren’t very healthy and what choice have they got about cooking…you go shopping with mum…and say, oh I’d like that and can we try that, but that’s quite hard for… 11, 12* *year old if mum is very, you know, right we’ll buy this, this and this, and you have this Monday, this Tuesday…”*
Child competency	*“Because there’s a bit of an issue there around giving competency to a 13* *year old isn’t there?”* *“…there’s an option [to challenge a parents refusal of HPV vaccine], … but with diet it would be slightly different isn’t it because… if the child wants to address health needs then there should be an option available to them”*
NCMP letter	*“I think there is a stigma attached to it, the fact that some children are getting ‘fat letters’ and in actual fact in that parents eyes their child hasn’t got a weight issue”* *“…I’ve had parents come and see me at drop ins or ring me up saying, who do you think you are saying my child is overweight? And it’s quite confrontational really”* *“But I think in a lot of ways it’s not really useful to our service that we are being sort of tarred with the nurses that call their children overweight, and it’s creating a bit of a barrier…”*
Closing the feedback loop	*“The only thing that I find sometimes a little bit frustrating is that you don’t have any feedback as to how well the programme went; did the family or child lose weight or didn’t they attend; that’s the only criticism I would have for the programme”* *“… sometimes it would be nice just to have like a letter to say, completed a 6*-*week programme or a 10*-*week programme and this is the outcome, really”* *“it would be nice to know, are they attending,…but we wouldn’t know the outcome unless it sort of comes back to us or we chase the family up to see whether they actually went, and what the outcome was at the end”*

School nurses discussed the difficulty of how to engage parents in supporting their children’s desire for making healthy changes. Little consensus was reached on an effective approach. There was a sense of helplessness conveyed in both FGs. There was a clear consensus that there would be little chance of acting on referrals and children making and sustaining healthy changes without family support.

#### Child autonomy

Most school nurses shared that children presenting issues appeared highly motivated to make changes regarding their weight, but expressed concerns as to how to instigate and maintain changes within their family dynamic. Children’s right to make decisions about their own bodies was identified as an important inconsistency. Namely between a child’s right to challenge their parent’s refusal of receiving the human papilloma virus (HPV) vaccine (used as an example in one FG), versus their lack of right to challenge a weight referral.

#### The National Childhood Measurement Programme

School nurses recognised the importance of the NCMP, but expressed concern over negativity surrounding its implementation. They discussed how the NCMP could make conversations with parents difficult suggesting it acts as a barrier to optimal communication between parents, children and school nurses. Second, nurses reported that NCMP data were not optimally utilised locally. Nurses discussed the data currently ‘standing alone’ and that contextualising NCMP data locally could be used as a facilitator for engaging school nurses in ongoing referral-based services.

#### Closing the feedback loop

School nurses consistently expressed a need for feedback from EWMM. Nurses described how feedback might improve their knowledge of EWMM and what other children and parents can expect, which could increase the likelihood of parental engagement.

#### GPs views

##### Knowledge of childhood obesity and EWMM

Two GPs interviewed had not referred into EWMM recently but all were aware of its predecessor programmes (Box 2). GPs expressed the importance of services such as EWMM feeding back whether a referral was taken up and if that service was completed (echoing school nurses).

GPs suggested how they would like to receive information about EWMM that would increase the chance of referral into the service:

- A visual prompt in the GP office such as a chart or characterisations of body shapes.
- More frequent face-to-face information sessions at their practice to keep them up-to-date with what services are available.
- Receipt of flyers and posters to put on practice notice boards or electronic screens in practices.
- Regular follow up and feedback on patient attendance, completion, drop out and outcomes.

##### Talking about child weight

Two GPs did not see addressing obesity as a problem and felt that parents were generally receptive when child overweight was raised. The other GPs interviewed described a hesitance to have this ‘difficult conversation’ with parents and children. GPs offered approaches for addressing children’s weight which included both parent-focused and child-focused ‘tactics’. GPs felt this was a distinctly different conversation than one had with adults and they needed to do it “carefully and subtly” (Box 2).

##### Box 2 GP views and experiences

**Table:** 

Topic	Quotation
Awareness of EWMM	*“Well to be honest with you I don’t know much about it because I was asking my… assistant practice manager about this and she’s always on the ball and she knows all the services. And … she hadn’t heard of it … if it is active we haven’t been using it”* *“[Interviewer] Okay. And have you referred any patients to…? [GP]: I haven’t personally,… but I’ve had patients who have been referred on my behalf”* *“I know it’s a referral system which I think patients can refer themselves, if I remember rightly, and it’s kind of to do with eating and exercise and management of weight problems, yes?… I think I was probably thinking about something slightly different from Eat Well because this was a couple of years ago…”* *“I think it probably could do with a bit of a re*-*launch with the general practice population. Because it’s a service you don’t use very often you tend to sort of forget it exists”*
The difficult conversation	*“…I think it’s a little bit more difficult really in children”* *“It is difficult yes to be perfectly honest. With adults it’s much, much, much easier. With children because when they come to see you they come with their parents and it’s just unfortunate sort of difficult discussion to start off with…”* *“If you just say, well he needs to lose weight, you know, that kind of will put barriers up straight away really”* *“So as a GP yes I do find it difficult to address it to be truthful”* *“But a child isn’t going to say, I do need to lose some weight. I think it usually comes from the parents. And I think unfortunately as I mentioned, often it tends to run in families for lots of reasons doesn’t it, so the parents are overweight, so they kind of won’t really necessarily perceive that the child is overweight. So I think it’s a little bit more difficult really in children”* *“The parents, you know, take the attitude, oh it’s just a phase they’ll grow out of, which a lot of them do, but the children are much more conscious about it I find”*
Approaches for addressing weight management in children	*“I think most adults and children if they are overweight they know it, and I think if you say to someone, you need to lose weight, it’s not going to help very much because they already know that. …So it has to be done quite, you know, kind of carefully and subtly really”* *“I think you listen to what the parents are saying and what… I mean one way about it is saying, well what are your views about it, and they might say well, I think he or she is a bit of overweight. And that can kind of bring it into the conversation sometimes”* *“So sometimes it maybe, you know, actually if you ask the child directly what they think about things rather than the parents really. So I think you have to kind of skirt around it a little bit but at the end of the day the child is the patient in front of you and they need to be…to get anywhere with treatment they need to be quite involved, well need to be fully involved with the process really”*

GPs discussed strategies of addressing weight with families (Box 2). Two GPs described a particular approach: shifting the conversation from presenting symptoms (such as asthma or joint pain) toward a focus on causes of the symptoms might be, how weight might affect them, and how losing weight might alleviate them.

##### Synthesis of findings

This study has identified two factors contributing to lower than expected referral rates into a community child weight management intervention, EWMM. First, a lack of knowledge exchange and feedback between service providers and referrers. Second, a resistance among health professionals to address child weight with parents and children, which we refer to as ‘the difficult conversation’.

The taboo of overweight (considered here as an avoidance of weight terminology and reluctance to engage individuals in conversations about weight) was observed in both school nurses and GPs. This is not a new or surprising finding given a recent emphasis on personal responsibility for weight management or the cultural politics regarding children’s weight [19–23]. Discussing overweight remains an issue fraught with emotional, psychological and physical risks, as well as benefits [24, 25].

For two GPs in this study, the taboo of discussing a child’s weight related to the hesitancy to have the difficult conversation between parents, health professionals, and children. Similarly, school nurses found that broaching the subject of obesity with parents was challenging and that they faced backlash from parents as a consequence. This difficult conversation has been identified in other populations as a barrier to referrals into weight management services [26–29].

Our study reflects a small set of health professionals’ views on the continued challenge of raising the issue of children’s weight, as well as how to manage that conversation between parents, children and health professionals [30–33]. Interventions which promote conversations between health professionals, children and families have shown some success [34–37]; suggesting improved confidence and skills among health professionals. However the most effective and sustainable interventions remain unclear [38]. A key insight from our conversation with school nurses suggested that children’s voice and autonomy merits greater consideration in approaches to accessing weight management among children and adolescents, particularly when their parents may hold different views to addressing their weight. This finding has been expressed elsewhere as important, illustrating a next step in maximising uptake of effective weight management interventions for children [32, 34, 38].

### Conclusion

Our study identified a complicated network of practice-level communication and feedback challenges and facilitators for a community-based child weight management intervention. This study contributes to evidence that low intervention uptake may be related to health professionals’ hesitancy to have difficult conversations with children and families.

#### Recommendations for practice and research

Future research could identify the extent to which health professionals report ‘the difficult conversation’ as a barrier to referral into child weight management services, and develop related training and communication strategies if warranted. Examining optimal training and communication strategies between families and health professionals that are more inclusive of children’s voice and autonomy also seems warranted.

## Limitations

This study was conducted among a small purposive sample of participants associated with a specific weight management service. It may not be applicable to services addressing child weight where intervention components or referral pathways differ substantially. Very few GPs responded to requests to discuss this topic, despite multiple attempts to contact them. This means data saturation may not have been reached and made it difficult to gauge the extent to which identified barriers are commonly perceived among the wider GP population in Solihull, and the UK.

## Abbreviations

BMI: body mass index
EWMM: Eat Well Move More
FG: focus group
GPs: general practitioners
HPV: human papilloma virus
NCMP: National Child Measurement Programme
NICE: National Institute for Health and Care Excellence
OSOP: ‘One Sheet of Paper’ technique

## References

[1] Robertson W, Murphy M, Johnson R (2016). Evidence base for the prevention and management of child obesity. Paediatr Child Health.

[2] Fat NL. Children’s body mass index, overweight, and obesity, chap 10. Health Survey for England, vol 1. 2014. http://content.digital.nhs.uk/catalogue/PUB19295/HSE2014-ch10-child-obe.pdf.

[3] Global strategy on diet, physical activity and health: childhood overweight and obesity strategy. World Health Organization; 2016. http://www.who.int/dietphysicalactivity/childhood/en/.

[4] Oyebode O, Mindell J (2013). Use of data from the Health Survey for England in obesity policy making and monitoring. Obes Rev.

[5] Oude Luttikhuis H (2009). Cochrane review: interventions for treating obesity in children. Evidencebased Child Health Cochrane Rev J.

[6] Colquitt JL, Loveman E, O'Malley C, Azevedo LB, Mead E, Al-Khudairy L, Ells LJ, Metzendorf MI, Rees K (2016). Diet, physical activity, and behavioural interventions for the treatment of overweight or obesity in preschool children up to the age of 6 years. Cochrane Database Syst Rev..

[7] Loveman E, Al‐Khudairy L, Johnson RE, Robertson W, Colquitt JL, Mead EL, Ells LJ, Metzendorf MI, Rees K. Parent-only interventions for childhood overweight or obesity in children aged 5 to 11 years. Cochrane Database Syst Rev. 2015;(12):CD012008. 10.1002/14651858.CD012008. 10.1002/14651858.CD012008 PMC8761478 26690844

[8] Obesity: identification, assessment and management. The National Institute for Health and Care Excellence (NICE); 2014. https://www.nice.org.uk/guidance/cg189/chapter/1-Recommendations. 36719951

[9] Aicken C, Roberts H, Arai L (2010). Mapping service activity: the example of childhood obesity schemes in England. BMC Public Health.

[10] Skelton J, Beech B (2011). Attrition in paediatric weight management: a review of the literature and new directions. Obes Rev.

[11] Ball GD (2012). Should I stay or should I go? Understanding families’ decisions regarding initiating, continuing, and terminating health services for managing pediatric obesity: the protocol for a multi-center, qualitative study. BMC Health Serv Res.

[12] Michie S (2008). From theory to intervention: mapping theoretically derived behavioural determinants to behaviour change techniques. Appl Psychol.

[13] Michie S, van Stralen MM, West R (2011). The behaviour change wheel: a new method for characterising and designing behaviour change interventions. Implement Sci.

[14] Measuring and interpreting BMI in children. Public Health England. 2012. http://webarchive.nationalarchives.gov.uk/20170110171049/https://www.noo.org.uk/NOO_about_obesity/measurement/children.

[15] Ells LJ (2015). Prevalence of severe childhood obesity in England: 2006–2013. Arch Dis Child.

[16] Stamatakis E, Wardle J, Cole TJ (2010). Childhood obesity and overweight prevalence trends in England: evidence for growing socioeconomic disparities. Int J Obes.

[17] Ziebland S, McPherson A (2006). Making sense of qualitative data analysis: an introduction with illustrations from DIPEx (personal experiences of health and illness). Med Educ.

[18] QSR (2012). NVivo qualitative data analysis software, Version 10.

[19] Casas C. Obesity discourse and fat politics: review of obesity discourse and fat politics: research, critique and interventions. In: Monaghan L, Colls R, Evans B, Editors. New York: Routledge; 2014. 2015, Taylor & Francis.

[20] Farrell LC (2016). Emotion in obesity discourse: understanding public attitudes towards regulations for obesity prevention. Sociol Health Illn.

[21] King LA (2007). Australian GPs’ perceptions about child and adolescent overweight and obesity the Weight of Opinion study. Br J Gen Pract.

[22] Mainland M, Shaw S, Prier A (2015). Fearing fat: exploring the discursive links between childhood obesity, parenting, and leisure. J Leisure Res.

[23] Toftemo I, Glavin K, Lagerløv P (2013). Parents’ views and experiences when their preschool child is identified as overweight: a qualitative study in primary care. Fam Pract.

[24] Katz A (2014). The last taboo?. Oncol Nurs Forum..

[25] Steele RG (2011). School nurses’ perceived barriers to discussing weight with children and their families: a qualitative approach. J School Health.

[26] Gerards SM (2012). Barriers to successful recruitment of parents of overweight children for an obesity prevention intervention: a qualitative study among youth health care professionals. BMC Fam Pract.

[27] Walker O (2007). A qualitative study of primary care clinicians’ views of treating childhood obesity. BMC Fam Pract.

[28] Teixeira FV, Pais-Ribeiro JL, Maia A (2015). A qualitative study of GPs’ views towards obesity: are they fighting or giving up?. Public Health.

[29] Zhu D (2015). Weight management care practices of English and Chinese nurses. Int Nurs Rev.

[30] Helseth S, Riiser K, Holmberg Fagerlund B, Misvaer N, Glavin K (2017). Implementing guidelines for preventing, identifying and treating adolescent overweight and obesity; school nurses’ perceptions of the challenges involved. J Clin Nurs..

[31] Nafiu OO, Chimbira WT, Tait AR (2018). Pediatric preoperative assessment: six million missed opportunities for childhood obesity education. Anesth Analg.

[32] Riiser K (2015). Targeting and tailoring an intervention for adolescents who are overweight: some ethical concerns. Nurs Ethics.

[33] Schalkwijk AA (2016). Health care providers’ perceived barriers to and need for the implementation of a national integrated health care standard on childhood obesity in the Netherlands—a mixed methods approach. BMC Health Serv Res.

[34] Moore SM, Killion CM, Andrisin S, Lissemore F, Primm T, Olayinka O, Borawski EA (2017). Use of appreciative inquiry to engage parents as codesigners of a weight management intervention for adolescents. Childhood Obes..

[35] Shue CK (2016). Promoting conversations between physicians and families about childhood obesity: evaluation of physician communication training within a clinical practice improvement initiative. Health Commun.

[36] van Mil E, Struik A (2017). Overweight and obesity in children: more than just the Kilos. Pediatr Phys Ther.

[37] Turner GL, Owen S, Watson PM (2016). Addressing childhood obesity at school entry: qualitative experiences of school health professionals. J Child Health Care.

[38] McPherson A (2017). Communicating with children and families about obesity and weight-related topics: a scoping review of best practices. Obes Rev.

